# From Bone Marrow Necrosis to Gaucher Disease; A Long Way to Run

**DOI:** 10.4274/tjh.2015.0123

**Published:** 2015-12-03

**Authors:** Neslihan Erdem, Ahmet Çizmecioğlu, İsmet Aydoğdu

**Affiliations:** 1 Celal Bayar University Faculty of Medicine, Department of Internal Medicine, Manisa, Turkey; 2 Karaman State Hospital, Clinic of Internal Medicine, Karaman, Turkey; 3 Celal Bayar University Faculty of Medicine, Department of Hematology, Manisa, Turkey

**Keywords:** Necrosis, Gaucher, Bone marrow

## TO THE EDITOR

Bone marrow necrosis (BMN) is a disease characterized with fever and bone pain and caused by many different malignancies, benign diseases and drugs. We reported a case of BMN due to diclofenac in 2006 [1]. And now we present the same patient with a corrected diagnosis, seven years after the first presentation.

**Figure 1:**
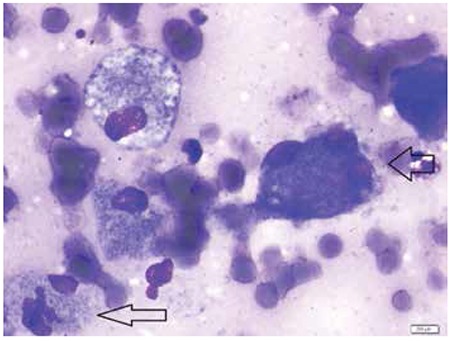
Gaucher cells, striated and fibrillary like cytoplasm with small dense nucleus.

A 26-year-old male presented with fever, bone pain, splenomegaly, anemia, leucopenia and was diagnosed with BMN due to diclofenac consumption. Nine months after his initial admission, his laboratory and physical examination were normal. Seven year after diagnosis, he was admitted to hospital due to bone pain. He had splenomegaly, leukocyte level was 6.22x109/L, hemoglobin level was 13.7 g/dL and thrombocyte level was 152x109/L. Because of history of BMN and reccurring splenomegaly, bone marrow aspiration and biopsy were performed. He was diagnosed with Gaucher disease in bone marrow biopsy and diagnosis was also confirmed by pathology. He had low glucosylceramide level (0.53 µkat/kg protein, normal range 2.4-3.8 µkat/kg protein) and high chitotriosidase level (2793 µkat/kg protein, normal range <40 µkat/kg protein). His treatment was started with imiglucerase. When we retrospectively revaluated the first bone marrow aspiration which had been made 7 years before, we saw that Gaucher cells were also present (Figure 1).

Gaucher disease is an autosomal ressesive, familial disease which presents with hepatosplenomegaly, skin pigmentation, bone lesions, anemia, leukopenia and thrombocytopenia. Interestingly, our patient was diagnosed with BMN and after 7 years, his diagnosis was corrected as Gaucher diesase with new bone marrow biopsy and reevaluation of the first bone marrow biopsy. BMN is characterized by necrosis of the medullary stroma and myeloid tissues in bone marrow, because of failure of microcirculation. Chemotherapy, microvascular infarction, tumor necrosis factor, and thrombosis were blamed for the pathophysiology [2]. It’s characterized with presence of amorphous esosinophilic material and ghost-like haematopoietic cells with irregular cell membranes. As seen in Figure 1, amorphous and corrupted appearance of the cells misled us. But when inspected carefully, typical Gaucher cells can be seen. Gaucher disease is characterized by accumulation of glucosylceramide in spleen, liver and bone marrow due to lysosomal glucocerebrosidase deficiency [3]. There is usually latency in diagnosis of Gaucher disease because of its rarity and not thinking of the disease in the first step but in further steps. In our patient, BMN due to diclofenac consumption was diagnosed in the first place, but after recurring splenomegaly and bone pain, he was reevaluated and diagnosed with Gaucher disease. Gaucher disease can be easily confused with other hematological diseases and can be overlooked. This leads us to the conclusion that diagnosis of a disease is a long way and if we have doubts about the diagnosis or unexpected changes are present, we should check the diagnosis and make further investigations. Gaucher disease must be kept in mind while making differential diagnosis in patients with splenomegaly, bone pain and pancytopenia.
